# An Introduction to The Royan Human Ovarian Tissue Bank 

**DOI:** 10.22074/ijfs.2016.4918

**Published:** 2016-06-01

**Authors:** Naeimeh Sadat Abtahi, Bita Ebrahimi, Rouhollah Fathi, Sepideh Khodaverdi, Abolfazl Mehdizadeh Kashi, Mojtaba Rezazadeh Valojerdi

**Affiliations:** 1 Department of Embryology, Reproductive Biomedicine Research Center, Royan Institute for Reproductive Biomedicine, ACECR, Tehran, Iran; 2 Endometriosis Research Center, Iran University of Medical Science, Tehran, Iran; 3 Department of Anatomy, Faculty of Medical Sciences, Tarbiat Modares University, Tehran, Iran

**Keywords:** Fertility Preservation, Human, Cancer, Ovarian Tissue Cryopreservation

## Abstract

From December 2000 until 2010, the researchers at Royan Institute conducted a wide range of investigations on ovarian tissue cryopreservation with the intent to provide fertility pres- ervation to cancer patients that were considered to be candidates for these services. In 2010, Royan Institute established the Royan Human Ovarian Tissue Bank as a subgroup of the Embryology Department. Since its inception, approximately 180 patients between the ages of 747 years have undergone consultations. Ovarian samples were cryopreserved from 47 patients (age: 7-35 years) diagnosed with cervical adenocarcinoma (n=9); breast carcinoma (n=7), Ewing’s sarcoma (n=7), opposite side ovarian tumor (n=7), endometrial adenocarci- noma (n=4), malignant colon tumors (n=3), as well as Hodgkin’s lymphoma, major thalas- semia and acute lymphoblastic leukemia (n=1-2 patients for each disease). Additionally, two patients requested ovarian tissue transplantation after completion of their treatments.

## Introduction

There is a concerning increase in cancer diagnoses according to the Iranian Cancer Society. Today, due to medical advances, many cancers are treatable with timely diagnosis and follow up. The patient can re- turn to a normal life after radiotherapy, chemothera- py, or surgical tumor excision. Therefore, many can- cers are no longer considered incurable. Although, in many cases, chemotherapy and radiotherapy aim to save lives, premature ovarian failure and reduction of follicular reserve is undeniable. By taking into con- sideration the probable infertility of cancer patients, preservation of their reproductive ability prior to onset of cancer treatment is crucial (1, 2). Different methods of assisted reproductive techniques that in- clude oocyte, embryo and ovarian tissue cryopreser- vation have helped these patients. The use of these techniques in single or married, as well as young and older women differ. Hence, the most appropriate technique is selected according to the patients’ cir- cumstances (1-7). In cases where adequate time exists for ovarian stimulation, embryo cryopreservation is considered the gold standard and an acceptable clini- cal technique. However, if embryo cryopreservation is not an option due to the absence of a sexual partner, unwillingness to use donor sperm, or for any other reason, the oocytes can be frozen (6). Ovarian tissue cryopreservation is another technique that has a long history of use, but with a new purpose. Limitations of oocyte cryopreservation exist, such as the impos- sibility of stimulating ovaries in patients with hyper- stimulation syndrome. Under these circumstances, ovarian tissue cryopreservation is more accepted and approved (2, 5). In this technique numerous follicles at different stages of maturity are preserved without delays to cancer treatment. In addition, for single or young girls this is the best choice to preserve their reproductive ability (3, 4). 

The Royan Human Ovarian Tissue Bank was es- tablished in 2010 with the intent to provide fertility preservation services to cancer patients eligible for preservation of reproductive ability. We have estab- lished the maximum age for inclusion in the Tissue Bank as 35 years. Cases of malignancy where tu- mors have metastasized to the ovarian tissue are not accepted for cryopreservation. In other cases there is no exclusion for acceptance. Patients undergo an ini- tial consultation that determines individual factors of age, marital status, physical and mental conditions, cancer type, its progression stage and grade, level of previous treatments, earlier infertility treatment and prognosis after treatment. After the initial consulta- tion, the best fertility preservation technique is se- lected. A contract is signed between the Ovarian Tis- sue Bank and the patient after the consultation. This contract includes patients’ rights and sample mainte- nance insurance, as well as informing patients about the use of her own sample after treatment, which is approved by the Royan Ethical Committee. 

**Fig.1:**
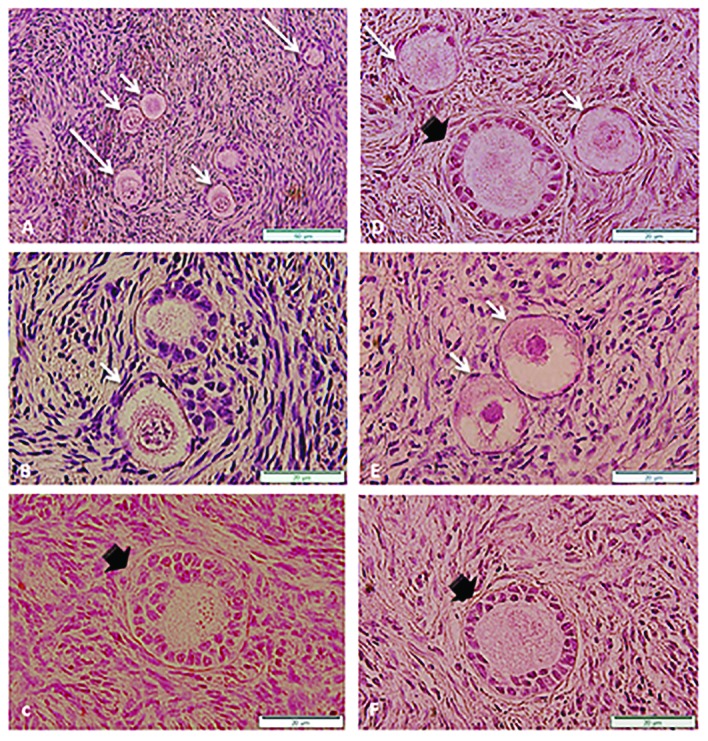
Primordial (short arrows), primary (long arrows) and preantral (black arrow) follicles in A., B., C. Control, D., E. and F. Vitrified hu- man ovarian tissues. Hematoxylin and eosin (H&E) staining (magnification: ×20, ×50 μm).

The procedure for ovarian tissue cryopreserva- tion at the Royan Human Ovarian Tissue Bank is as follows. An ovarian tissue sample is removed from the patient by laparoscopy, laparotomy, unilateral or bilateral oophorectomy according to the patient’s condition. The sample is transferred to the Ovarian Bank in the shortest possible time (approximately 1 hour) in Medium 199+Heppes (HTCM, Gibco, Paisley, UK)+20% human serum albumin (HAS, Biotest, Germany) as transfer medium at 4°C and on ice. In the laboratory, initially, the transferred tissue is washed in HTCM+20% HSA medium, after which the medullary part is removed. Next, the cortical part is thinned and 10×5×1 mm strips are obtained from the thin cortex. These steps are all performed on a cool pad. Finally, the stripes are vitrified in a two-step process, equilibration and vitrification. In the first step (equilibration), each strip is washed in equilibrium medium composed of HTCM, ethylene glycol (EG, Sigma, St. Louis, MO, USA), Dimethyl sulphoxide (DMSO, Sigma, USA, each 7.5%) and 20% HSA for 15 minutes at 4°C. In the second step (vitrification), each strip is washed in 15% HTCM, 15% DSMO and 15% EG, 0.25 M sucrose, and 20% HSA for 10 minutes at 4°C. The extra medium is completely removed from the strips, after which they are directly trans- ferred into liquid nitrogen. Of note, we randomly fix one strip before cryopreservation for histologi- cal evaluation (H&E staining and Semi thin). For tissue evaluation, one vitrified strip is warmed and assessed histologically. Warming is performed in 4 steps in descending concentrations (1, 0.5, 0.25, and 0.125) of sucrose. The base medium is com- prised of HTCM+20% HSA. The histological as- sessment markers considered for tissue evaluation include total integrity, follicular population, oo- cyte degeneration, vacuolization and granulation of the nucleus, oolemma and ooplasm conditions, zona pellucida situation (in secondary or preantral follicle), coherence and connectivity of granulosa cells (Fig .1). 

Functionality of the entire ovarian tissue is con- sidered by the presence or absence of the corpus luteum or corpus albicans in tissue. Finally, the in- formation is kept and filed in a histology descrip- tion form.
